# Heat Production of Iberian Pig Exposed to High Temperature and Effect of Dietary Supplementation with Betaine or Zinc

**DOI:** 10.3390/ani14142033

**Published:** 2024-07-10

**Authors:** Manuel Lachica, Zaira Pardo, Luis Lara, Rosa Nieto, Ignacio Fernández-Fígares

**Affiliations:** Department of Nutrition and Sustainable Animal Production, Estación Experimental del Zaidín, CSIC, San Miguel 101, 18100 Armilla, Granada, Spain; zaira.pardo@eez.csic.es (Z.P.); luis.lara@eez.csic.es (L.L.); rosa.nieto@eez.csic.es (R.N.); ignacio.fernandez-figares@eez.csic.es (I.F.-F.)

**Keywords:** betaine, environment, heat production, heat stress, pig, zinc

## Abstract

**Simple Summary:**

As pigs are sensitive to heat, high temperature produces large economic losses in pig production. The Iberian pig (*Sus mediterraneus*) is a rustic breed that thrives in the Mediterranean forest in the southwest of the Iberian Peninsula, with hot summers, with a peak in July (32–36 °C) being more frequent due to the appearance of heatwaves in the Mediterranean area. It is well known that betaine and zinc are growth promoters in pigs under thermoneutral conditions. Betaine acts as an osmolyte, decreasing basal heat production, and zinc is very important as an enzymatic cofactor and structural element for proteins, and improves intestinal functionality. Whether betaine and zinc may mitigate heat stress in pigs remains unclear. Our aim was to study the effect of heat, and the dietary supplementation of betaine or zinc on the heat production of Iberian pigs. At 30 °C, betaine or zinc supplementation had no effect on heat production; in addition, these parameters were not affected by temperature, indicating that Iberian pigs are well adapted to hot environments. However, the ratio of CO_2_ production/O_2_ consumption indicated that supplementation with betaine may have a positive effect on lipogenesis and, thereafter, overall growth.

**Abstract:**

The effect of heat, and dietary betaine or zinc on the heat production (HP) of Iberian pigs was studied. Thirty barrows (44 kg) were individually housed for 28 days and assigned to one of five treatments: (1) thermoneutrality (20 °C) and fed a control diet (TN-CON) ad libitum; (2) hot (30 °C) and fed a control diet (HT-CON) ad libitum; (3) thermoneutrality and pair fed a control diet (TN-CON-PF) to HT-CON; (4) hot and fed a betaine-supplemented (0.5%) diet (HT-BET) ad libitum; and (5) hot and fed a zinc-supplemented (0.012%) diet (HT-ZN) ad libitum. On the 18th day, pigs were moved to a respirometry chamber (two chambers) under their respective treatment. The metabolizable energy (ME) intake, HP and respiratory quotient (RQ) were measured over 24 h. No differences (*p* > 0.05) were found in HP and RE between treatments. For RQ, TN-CON was greater (*p* < 0.01) than HT treatments, except for HT-BET. All RQs indicated an overall lipogenesis where betaine supplementation showed an intermediate value, indicating that it may have a positive effect on lipogenesis and overall growth. At 30 °C, betaine or zinc had no effect on HP and RE; ME intake was not reduced, indicating a genetic adaptation of Iberian pigs to heat.

## 1. Introduction

High temperature produces major economic losses in pig production as a result of reduced growth rate, altered carcass traits and increased mortality, among others [1]. The main pig producing areas on the Iberian Peninsula (Spain and Portugal) are under a Mediterranean climate with hot summers, with a peak in July (32–36 °C) being more frequent due to the appearance of heatwaves in the Mediterranean area.

Pigs are sensitive to heat, activating thermoregulatory responses above 25 °C [2]. Nonetheless, the Iberian pig is an autochthonous non-selected pig breed that is very rustic and adapted to its environment [3]. On the other hand, pigs selected for low residual feed intake (more efficient) might be more robust and resilient than pigs selected for high residual feed intake (less efficient [4]), as the more-efficient low-residual-feed-intake pigs reduce the total amount of heat production (HP) by the unit of metabolizable energy (ME) intake.

Betaine [5] and zinc [6] have been used as growth promoters in pigs under thermoneutral conditions. Betaine acts as an osmolyte, decreasing basal HP and, therefore, ME for maintenance [7]. Zinc is very important as an enzymatic cofactor and structural element for proteins [8]; it increases total hemoglobin, the red blood cell count and hematocrit via the hormone erythropoietin, enhancing the O_2_ carrying capacity [9]. In addition, it improves intestinal functionality [10]. They may both be useful to reduce the effect of heat load in heat-stressed pigs. However, whether betaine and zinc may mitigate heat stress in pigs remains unclear; it is not known how heat may affect HP, and therefore the energy utilization of Iberian pigs, in spite of the more frequent heat spikes in the Mediterranean area.

Our aim was to study the effect of heat, and the dietary supplementation of betaine or zinc on the HP (and the retained energy (RE)) of Iberian pigs.

## 2. Materials and Methods

### 2.1. Animals, Treatments and Diets

Experimental procedures and animal care were in agreement with Spanish Ministry of Agriculture guidelines (RD53/2013). The Bioethical Committee of the CSIC (Spanish Council for Scientific Research, Spain) and the competent authority (Junta de Andalucía, Spain, project reference 28/06/2016/118) approved all the experimental procedures with the animals used in the present study.

Thirty pure Iberian barrows (Sánchez Romero Carvajal strain; Sanchez Romero Carvajal Jabugo S.A. (Puerto de Santa María, Cádiz, Spain)) of 44 ± 0.97 kg initial body weight (BW) were used. Pigs were randomly assigned to one of the five environmental and dietary conditions (six pigs per treatment): (1) thermoneutral temperature (20 °C) and fed a control diet (TN-CON) ad libitum; (2) hot temperature (30 °C) and fed a control diet (HT-CON) ad libitum; (3) thermoneutral temperature and pair fed a control diet (TN-CON-PF) to HT-CON (to eliminate confounding effects of a dissimilar feed intake); (4) hot temperature and fed a betaine-supplemented diet (HT-BET) ad libitum; and (5) hot temperature and fed a zinc-supplemented diet (HT-ZN) ad libitum. Pigs were individually housed in 2 m^2^ slatted pens, allowing visual contact among them and with free access to water at all times. Experimental conditions were maintained for 28 days. Diet composition is displayed in Table 1.

Three diets were used. The control diet was barley–soy bean meal based (146 g crude protein/kg and 12.3 MJ ME/kg) with an optimal amino acid profile [12] and covering all nutrient requirements [13]. The betaine diet was supplemented with 5 g/kg betaine (TNI-Betain, natural origin, anhydrous, 960 g/kg purity; Trouw Nutrition-Nutreco, Madrid, Spain) and the zinc diet was supplemented with 120 mg/kg zinc sulfate (ZnSO_4_ × H_2_O, 980 g/kg purity; VWR, Leuven, Belgium). Feed allowance for TN-CON-PF group was calculated daily based on the HT-CON group’s intake the previous day. Daily intake was recorded.

During the 28-day experimental period, barrows were allocated to one of two environmentally controlled rooms and received the appropriate diet according to the treatments. The temperature was progressively raised for HT treatments from 20 to 30 °C, and controlled using an air conditioning apparatus (LG UM36, LG Electronics Inc., Changwon, Republic of Korea). The temperature and relative humidity was recorded every 15 min by a data logger (HOBO UX100-011; Onset Computer Corporation, Bourne, MA, USA). The photoperiod was fixed to 12 h of light (08:00 to 20:00 h) and 12 h of darkness. Three replications with two pigs per treatment were carried out.

On the 18th experimental day, pigs (61 ± 3.1 kg BW) were moved from their own slatted pens to one of the two open-circuit-type respirometry chambers, maintaining their respective environmental conditions. After an adaptation day, ME intake and total HP were measured over 24 h from O_2_ consumption and CO_2_ production according to Brouwer [14]. The respiratory quotient (RQ) was determined as the ratio of CO_2_ produced/O_2_ consumed. Periodic calibration of the whole system was carried out by injecting pure gases (CO_2_ (99.998%) and O_2_-free N_2_ (to produce an O_2_ decrement)) into the chamber, and calibration factors obtained were used for correcting CO_2_ production and O_2_ consumption, respectively.

**Figure 1:**
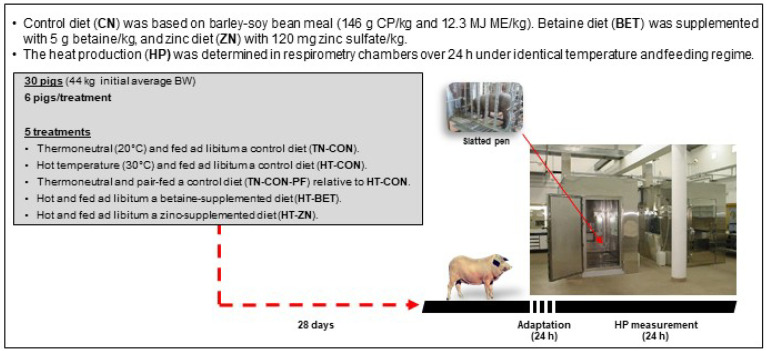
Experimental design.

The experimental design is displayed in Figure 1.

### 2.2. Chemical Analysis

Chemical analysis of the control diet is shown in Table 1. Samples of feeds were pooled along the experiment and analyzed in triplicate for dry matter (no. 934.01), ash (no. 942.05) and ether extract (no. 920.39) by standard procedures [15]. Total N was determined according to the Dumas’ method, by total combustion in TruSpec CN equipment (Leco Corporation, St. Joseph, MI, USA) and crude protein calculated as total N × 6.25. The neutral, acid and lignin detergent fractions (aNDFom (NDF assayed with a heat stable amylase and expressed not including residual ash), ADFom (ADF expressed not including residual ash) and Lignin(sa) (lignin determined by solubilization of cellulose with sulfuric acid), respectively) were analyzed by the method of Goering and van Soest [16]. Neutral and acid detergent fiber were determined using an ANKOM220 Fiber Analyzer Unit (ANKOM Technology Corporation, Macedon, NY, USA). Gross energy was measured in an isoperibolic bomb calorimeter (Parr Instrument Co., Moline, IL, USA).

### 2.3. Statistical Analysis

The number of animals (6/treatment, *n* = 30) was calculated using the G*Power software (Version 3.1.9.7; Heinrich-Heine-Universität Düsseldorf [17]), accepting an alpha risk of 0.05 and a beta risk of 0.2 in a two-sided test. The treatment effect was evaluated using the GLM procedure of SAS that included the fixed effects of treatment (TN-CON, TN-CON-PF, HT-CON, HT-BET and HT-ZN) and replication. As the replication effect was not significant, it was withdrawn from the model (completely randomized design). The animal was considered as a random effect. Differences (*p* < 0.05) were assessed using Tukey’s multiple-range test. The BW was used as the covariate.

## 3. Results

Average temperatures in the environmentally controlled rooms were 19.9 ± 0.20 and 30.2 ± 0.20 °C for the TN and HT treatments, respectively; relative humidity was 56.6 ± 0.17 and 55.6 ± 0.12% for the TN and HT treatments, respectively. The average temperatures in the respirometry chambers were 20.0 ± 0.01 and 30.0 ± 0.01 °C for the TN and HT treatments, respectively; relative humidity was 62.6 ± 0.12 and 62.3 ± 0.18%, respectively.

No differences (*p* > 0.05) were found in ME intake, HP and RE between treatments (Table 2). The treatment did not affect the RQ of pigs exposed to HT, it was greater (10.2%; *p* < 0.01) for TN-CON than the HT-CON and HT-ZN treatments.

## 4. Discussion

The world global rise in temperature represents a problem in animal production. Studies dealing with environmental heat effects on autochthonous breeds are scarce. One of these breeds is the Iberian pig, a rustic, indigenous, obese breed with a lower protein deposition and growth rate compared to modern breeds [12]. The thermoneutral zone of pigs depends on various factors but it could be established between 18 and 25 °C, and temperatures above 25 °C activate thermoregulatory responses [2]. Pigs under HT treatments in the present experiment were exposed to a long-term constant temperature (30 °C) for 28 days. No information about an upper critical temperature is available for Iberian pigs (or other non-improved breeds). The upper limit was chosen because it is close to the average temperature in the hottest months in the main Iberian pig-producing areas (south of Portugal and southwest Spain) where highs above 30 °C are only occasionally reached. The upper critical temperature is defined as the effective ambient temperature where HP is not offset by net heat loss to the environment without the aid of special heat-dissipating mechanisms, and the animal passes from a warm zone (thermoneutral zone) to heat stress. Huynh et al. [18] reported a variable upper critical temperature in pigs depending on the parameter studied, establishing, for instance, 25.5 °C when referring to HP. Although the Iberian pig is well adapted to the environmental conditions, spending time in open-range grazing even under strong sunshine and a high temperature, no data about their upper critical temperature is available in the literature.

Data of the present study on growth performance and plasma parameters have been published previously [11]. Briefly, heat increased rectal temperature and decreased feed intake. Weight gain in the TN-CON group was greater than with TN-CON-PF and HT-CON treatments. Temperature did not affect the gain-to-feed ratio. Betaine or zinc supplementation did not prevent the negative effect of heat on the growth performance of Iberian pigs. Fernández-Fígares et al. [19] reported that 0.5% dietary betaine produced the greatest differences in growth and body composition in young feed-restricted pigs over control pigs. We would like to mention that the dose of 5 g betaine/kg was widely used in previous studies carried out in our laboratory with good results and it was chosen for comparative purposes. The zinc dose was chosen because although only doses above 1000 mg of zinc/kg improved the growth performance of weaned pigs, the use of supplemental zinc at pharmacological doses is not allowed in the EU due to environmental concerns (150 mg/kg is the upper limit for swine).

We are aware that there may be a time effect for when the animals are in a treatment chamber and their entry into the respiratory chamber, since having two respiratory chambers means that not all animals enter at the same time during treatment. We try to compensate for this according to the following scheme: TN-CON and TN-CON-PF, HT-CON and HT-BET, HT-CON and HT-ZN, sequentially and successively, until the determinations are complete. Heat production showed no difference (*p* > 0.1) between the treatments, indicating no apparent effect of chronic heat stress, and no effect of betaine or zinc supplementation under heat stress conditions. However, rectal temperature was greater (*p* < 0.05) in the HT-CON treatment group compared to with the TN treatments and neither betaine nor zinc mitigated the increase in rectal temperature [11]. There is an adaptation to a hot environment: the thermoregulatory response in pigs comprises hyperthermia within the first 24 h of exposure to heat and a posterior recovery period with a gradual decrease in body temperature [2], which may explain the differences between acute and chronic heat stress studies.

When pigs are exposed to heat stress, feed intake (ME intake) is reduced [20,21] to decrease HP and, consequently, the amount of nutrients available for growth is lowered [22]. However, unlike improved breeds, ME intake and, consequently, growth (RE) in Iberian pigs were not reduced (*p* > 0.1) at 30 °C, which may be due to a genetic adaptation to harsh environments [3]. Furthermore, supplementation with betaine or zinc did not affect ME intake in the conditions of the current study (*p* > 0.1). Pardo et al. [11] reported a reduction in voluntary feed intake in Iberian pigs under HT conditions but to a lesser extent than in lean pigs. From data shown in Table 2, a paradoxical effect could be derived: numerically, the greater RE value corresponds to the HT-CON treatment, which had the lowest HP value, so the ME intake/HP ratio was higher than with the other treatments. This may indicate that Iberian pigs under an elevated temperature had the capacity to maintain their feed intake without increasing the HP associated with the heat increment of feed, which could be an adaptation to the imposed elevated temperature. Hao et al. [21] reported an increase in water consumption to reduce the heat increment of feeding in crossbred pigs under heat stress. Unfortunately, water consumption was not recorded in the present study.

Betaine or zinc supplementation had no effect on ME intake, HP or RE. Mendoza et al. [23] did not find an improvement in growth educed by betaine in late finishing pigs under heat stress under commercial conditions (32 °C for 6 weeks). Nonetheless, betaine elicited a slight increase in average daily gain but no change in feed efficiency in 25 kg BW crossbred pigs subjected to cyclical heat stress (37 °C for 9 h for 18 weeks) compared to pair-fed TN pigs [24]. Pardo et al. [11] found no improvement in Iberian pigs’ growth with betaine supplementation (30 °C for 28 days).

Zinc supplementation did not alter growth parameters in Iberian pigs [11] or in crossbred gilts (50 kg BW) under cyclical heat stress conditions for 7 days [25]. A higher level of zinc supplementation (1500 mg/kg) reduced BW loss in 11 kg BW Bama miniature pigs after short-term heat stress (40 °C for 5 h and 8 days [9]). However, in EU countries supplemental zinc over 150 mg/kg is not allowed for swine [26].

A way to reduce HP under HT conditions is to decrease visceral mass relative to BW. Pardo et al. [11] reported a numerical reduction in visceral mass relative to BW in Iberian pigs under HT conditions although it did not attain statistical significance. Additionally, Le Bellego et al. [27] reported a decrease in the total viscera weight in heat-stressed crossbred pigs. It is important to highlight that heat stress (33 vs. 23 °C [28]) may decrease visceral blood flow without noticeable changes in organ weight compared to pair-fed thermoneutral counterparts. Morales et al. [29] pointed out a reduction in the blood flow to the small intestine of heat-stressed pigs, decreasing the intestinal villus height. Iberian pigs show greater feed intake capacity compared to the Landrace genotype [30]; however, when feed intake was equal between both breeds under thermoneutral conditions, Iberian pigs showed lower portal-drained viscera HP and contribution of portal-drained viscera to whole HP than Landrace pigs [31], which can be an advantage in adapting to hot conditions.

Of note, the RQ value was greater (10.2%; *p* < 0.01) for TN-CON vs. HT-CON and TN-CON vs. HT-ZN. As it is known, RQ ranges from 0.7 to 1.0 when the animal uses lipids or carbohydrates as metabolic fuel, respectively. When the ME intake is ad libitum, lipids are synthesized from carbohydrates and the RQ value is greater than 1 (indicating low lipogenesis and/or high lipogenesis), implying better performance of the pigs. All RQ values indicated overall lipogenesis (RQ > 1) that was less active under the HT treatments, with no effect of betaine or zinc supplementation. Indeed, de novo lipogenesis (Acetyl-CoA-carboxylase activity) in the back fat, leaf fat and liver was less active in heat-stressed pigs compared to their pair-fed thermoneutral counterparts [32]. However, the supplementation with betaine showed an intermediate RQ value—between the TN and HS treatments—indicating that it may have a positive effect on lipogenesis and overall growth. Rojas-Cano et al. [33] reported a reduction in portal blood flow, O_2_ consumption and, therefore, portal-drained viscera HP in Iberian pigs fed a diet supplemented with betaine with respect to a control diet, potentially increasing the energy availability for other body tissues. A potential reduction in portal-drained viscera O_2_ consumption may help explain the lack of changes in RE in the HT-BET treatment group.

No change in RE does not necessarily imply no change in the RE partition as protein and fat. Protein deposition decreased in growing modern pigs reared at 30 °C compared with 23 °C [27]. This is in accordance with increments in blood urea N in crossbred finishing pigs under heat stress (35 °C for 7 days) compared with pair-fed animals under TN conditions (20 °C for 7 days [34,35]). Nevertheless, short-term heat stress did not affect post-absorptive urea (daily cyclical 23.6–37.6 °C for 21 days [36]) or fasting urea (33 °C for 21 days [37]). Pardo et al. [11] reported a tendency to increase fasting plasma urea N in HT in the Iberian pigs of the present experiment compared to pigs under pair-fed TN conditions, maybe indicating increased protein catabolism (decreased efficiency of N utilization), which is usually increased during chronic heat stress [22]. In addition, Pardo et al. [11] reported no changes in mesenteric fat as a percentage of empty BW, which is a proxy of visceral fat; plasma total protein was not different between treatments with only a tendency for a lower urea content under pair-fed TN conditions. Overall, it may be speculated in the present study that RE as fat was not affected as well as the RE as protein. In addition, in this study, betaine or zinc supplementation did not improve growth under heat stress conditions, or affect weights or relative weights of viscera or carcass weight.

## 5. Conclusions

At 30 °C, betaine or zinc supplementation had no effect on HP and RE; in addition, these parameters were not affected by temperature, indicating the effective adaptation of Iberian pigs to hot environments. Although not conclusively, the RQ values indicated that supplementation with betaine may have a positive effect on lipogenesis and, thereafter, overall growth. Further research is warranted to clarify the action of betaine with temperatures above 30 °C in the Iberian pig.

## Figures and Tables

**Table 1:** Composition and chemical analysis of the control diet ^1^.

Ingredients	
Barley grain, g/kg as fed	700
Corn, g/kg as fed	143.7
Soybean meal, g/kg as fed	127
Calcium phosphate, g/kg as fed	9.3
Calcium carbonate, g/kg as fed	6.2
Sodium chloride, g/kg as fed	3.0
L-Lysine (50%), g/kg as fed	5.0
L-Threonine (50%), g/kg as fed	2.1
Methionine hydroxy-analog (75%), g/kg as fed	0.7
Vitamins and minerals ^2^, g/kg as fed	3.0
Chemical analysis	
Dry matter, g/kg as fed	899
Ash, g/kg as fed	48.6
Ether extract, g/kg as fed	17.5
Crude protein, g/kg as fed	145.9
aNDFom ^3^, g/kg as fed	140.7
ADFom ^4^, g/kg as fed	44.5
Lignin(sa) ^5^, g/kg as fed	2.1
Gross energy (MJ/kg)	16.6

^1^ Pardo et al. [11]. ^2^ Provided (per kg of diet): 2000 UI retinol as retinyl acetate, 800 UI cholecalciferol, 40 UI dL-α-tocopheryl acetate, 1.5 mg menadione, 2 mg thiamine, 3 mg riboflavin, 50 μg cyanocobalamin, 15 μg folic acid, 22.5 mg nicotinic acid, 15 mg d-pantothenic acid, 60 mg MnO, 80 mg FeCO_3_, 80 mg ZnO, 750 μg KI, 10 mg CuSO_4_ × 5H_2_O, 50 μg Na_2_SeO_3_, 250 mg sepiolite, 1.5 mg butylhydroxyanisole (BHA) and 7.5 mg butylhydroxytoluene (BHT). ^3^ aNDFom, neutral detergent fiber inclusive of residual ash. ^4^ ADFom, acid detergent fiber not including residual ash. ^5^ Lignin(sa), lignin determined by solubilization of cellulose with sulfuric acid.

**Table 2:** Average body weight (BW), daily metabolizable energy (ME; kJ/kg^0.75^ BW) intake, heat production (HP; kJ/kg^0.75^ BW), retained energy (RE; kJ/kg^0.75^ BW), CO_2_ production (L/kg^0.75^ BW), O_2_ consumption (L/kg^0.75^ BW) and respiratory quotient (RQ; CO_2_/O_2_) in Iberian pigs (*n* = 6/treatment) fed ad libitum with a control (CON) diet supplemented either with betaine (BET; 5 g/kg) or zinc (ZN; 120 mg/kg) under thermoneutral (TN; 20 °C) or hot (HT; 30 °C) temperature.

	TN-CON	HT-CON	TN-CON-PF ^1^	HT-BET	HT-ZN	SEM	*p*-Value
BW	64.2	60.8	58.6	64.9	56.0	3.30	0.907
ME intake	1786	1707	1585	1715	1651	56.0	0.832
HP	976	792	830	879	851	30.8	0.411
RE ^2^	811	915	755	837	799	35.5	0.689
CO_2_	49.9	38.9	42.5	43.9	41.7	1.32	0.143
O_2_	42.1	36.0	36.3	39.4	38.7	1.12	0.418
RQ	1.19 ^a^	1.08 ^b^	1.17 ^ab^	1.11 ^ab^	1.08 ^b^	0.010	0.009

^1^ Thermoneutral and pair fed (PF) a control diet relative to HT-CON; ^2^ Calculated as RE = ME intake—HP; ^ab^ Values within a row with unlike superscript significantly differ (*p* < 0.01).

## Data Availability

The data presented in this study are available on request from the corresponding author.
